# Estimating age of menopause in mothers in the ALSPAC Study: A data note

**DOI:** 10.12688/wellcomeopenres.24760.2

**Published:** 2026-06-25

**Authors:** Rochelle Knight, Abigail Fraser, Carol Joinson, Ana Goncalves Soares

**Affiliations:** 1 MRC Integrative Epidemiology Unit, Bristol, England, UK; 2 Population Health Sciences, University of Bristol Medical School, Bristol, England, UK

**Keywords:** ALSPAC, menopause, age, longitudinal cohort

## Abstract

Earlier menopause is associated with increased risks of type 2 diabetes, osteoporosis, heart disease, depression, and mortality. Although menopause is typically defined as the absence of menstruation for 12 consecutive months, deriving age at natural menopause using repeated, prospective data can be challenging. In this data note, we describe our approach developed to assign age at natural menopause using longitudinal data from the Avon Longitudinal Study of Parents and Children (ALSPAC) Mothers cohort and highlight the complexities involved. Between 2008 and 2020, women were asked at up to eight timepoints about their menstrual history, including when they last had a menstrual period (LMP), reasons for period cessation if relevant, and contraception and hormone replacement therapy (HRT) use. Our approach used these repeated menstrual reports to assign the timing of the final menstrual period. Specifically, we combined three key variables - self-reported LMP and menstruation in the last 3 months and last 12 months – and accounted for factors affecting menstruation such as surgery, contraception, and HRT. Age at natural menopause was then derived using these repeated reports across timepoints. Of 5,949 women included in the analysis (mean 3.6 timepoints per participant), age at natural menopause was assigned for 2,422 women. The mean assigned age of menopause was 49.4 years (SD = 4.1, range: 30–63 years, median = 50 years). This data note introduces potential users of the ALSPAC data to our derived menopause variable and highlights key challenges when using longitudinal data to estimate menopause timing including irregular bleeding, missing data, and conflicting reports. Our aim is to support researchers wishing to use this derived variable in future studies of reproductive ageing.

## Background

Menopause is defined as the permanent cessation of menstruation, marking the loss of ovarian follicular activity. ^1, 2^ It occurs with the final menstrual period (FMP) and is typically diagnosed retrospectively after 12 months of amenorrhoea. ^1, 3^

Age at menopause is a marker of aging and overall health. A later age at menopause has been associated with increased life expectancy ^4^ and reduced all-cause mortality, ^5^ as well as a lower risk of cardiovascular disease ^4, 6– 12^ and osteoporosis. ^13, 14^ It is also linked to a higher risk of breast, ^15, 16^ endometrial, and ovarian cancers. ^4, 17– 20^

Previous studies have mostly relied on retrospective self-reports of age at FMP, which are susceptible to recall error, particularly when many years have passed since the menopause. ^21, 22^ While prospective data on menstrual cycles can help capture the variability in menstrual patterns and reduce recall error, accurately identifying the age at menopause using repeated, prospective data can pose challenges.

In this data note, we describe our approach to estimating the age at natural menopause using repeated menstrual cycle data from participants in a UK birth cohort.

## Rationale

In ALSPAC, women in the mothers cohort were asked their age at menopause in one questionnaire (Questionnaire U). This questionnaire was completed by 4,423 women at a mean age of 49.7 (SD 4.5). Of these, 1,616 reported their age of menopause (mean 48.65, SD 4.0), 2,536 women reported that they had not yet been through the menopause, and the remaining responses were missing.

Previous studies have derived the timing of the final menstrual period (FMP) in ALSPAC ^23– 25^; however, detailed descriptions of the derivation methods have generally not been reported. These studies excluded women who reported hormonal medication use at any time, rather than excluding only the specific timepoints where use was reported. They also did not incorporate the self-reported age at menopause variable. In addition, earlier approaches relied primarily on clinic data, as these were linked to the outcomes being analysed, despite questionnaire data also collecting information on menstrual bleeding history.

The method presented here was developed to provide a transparent and reproducible approach to estimating the FMP in ALSPAC. Our approach builds on previous work but refines the derivation by incorporating information from both questionnaires and clinic assessments and by making use of multiple questions relating to menstrual bleeding history. The approach also draws on guidance from the Stages of Reproductive Aging Workshop (STRAW) framework to inform the classification of menopausal status. We explicitly consider inconsistencies between responses and outline a systematic approach to handling missing data.

By documenting this approach, we aim to provide a consistent FMP variable that can be used by future researchers working with ALSPAC data. The approach is designed to be flexible: it can be extended as additional data becomes available. This approach can also be useful for other studies with prospective information on menstrual bleeding history, which may encounter similar challenges.

## Methods

### Participants

The Avon Longitudinal Study of Parents and Children (ALSPAC) is a longitudinal birth cohort that recruited pregnant women with an expected date of delivery between April 1991 and December 1992 residing in Avon, UK. The initial ALSPAC sample consisted of 14,541 pregnancies, from 14,203 unique mothers (338 mothers having two enrolled pregnancies), which resulted in 14,062 live births. As a result of the additional phases of recruitment, a further 630 mothers who did not enrol originally have provided data since their child was 7 years of age. This provides a total of 14,833 unique mothers (known as the Generation 0 or G0) enrolled in ALSPAC.

Since recruitment, mothers, their children and partners have been followed-up through questionnaires, research clinic assessments and data linkage. Since 2014, study data have been collected and managed using REDCap (Research Electronic Data Capture), a secure, web-based platform hosted at the University of Bristol. ^26^ REDCap is specifically designed to support data capture and management in research studies.

Details on the representativeness, cohort profiles and recruitment have been extensively described in previous publications. ^27– 30^ The ALSPAC study website contains details of all data available through a fully searchable data dictionary and variable search tool ( http://www.bristol.ac.uk/alspac/researchers/our-data ). In this study prospective data from ALSPAC G0 mothers was used.

### Ethical approval and consent

Ethical approval for the ALSPAC study was obtained from the ALSPAC Law and Ethics Committee and local research ethics committees. These approvals cover all core study data collection, including the questionnaires and clinic data that are used in the present analysis. All questionnaire content was reviewed and approved by the ALSPAC Ethics and Law Committee.

Initial approvals for the establishment of the cohort were granted by: Bristol and Weston Health Authority (E1808, *Children of the Nineties: Avon Longitudinal Study of Pregnancy and Childhood (ALSPAC))*, approved 28th November 1989; Southmead Health Authority (49/89, *Children of the Nineties – “ALSPAC”*), approved 5th April 1990; and Frenchay Health Authority (90/8, *Children of the Nineties*), approved 28th June 1990. Approval details for all subsequent clinics (committee, approval number, dates) are available here: https://www.bristol.ac.uk/media-library/sites/alspac/documents/governance/Research_Ethics_Committee_approval_references.pdf

Informed consent for the use of all data was obtained from participants in accordance with the recommendations of the Ethics and Law Committee at the time. The completion of a questionnaire, either on paper or online, was considered to be written consent from participants to use their data for research purposes. Participants can contact the study team at any time to retrospectively withdraw consent for their data to be used. Study participation is voluntary and during all data collection sweeps, information was provided on the intended use of data. Full details of the ALSPAC consent procedure are available on the study website.

In addition to these study-level approvals, researchers are required to submit individual level project proposals for consideration by the ALSPAC Executive Committee. The present project received such approval before data access was granted.

## Data available on menstrual cycles

Women were asked a set of detailed questions about their menstrual cycles including when they last had a menstrual period (LMP), reasons for period cessation if relevant, and their use of contraception and hormone replacement therapy (HRT), at eight timepoints, via postal questionnaires or in-person clinic assessments. Table 1 summarises the data collection timepoints, the number of questionnaire responses or clinic attendees, the year of completion, and the mean age at each timepoint. The average age at completion ranged from 47.4 (standard deviation [SD] 4.5) to 57.7 years (SD 4.5). A complete list of the questions and response options used to determine women’s date of LMP at each timepoint, i.e. their most recent menstrual period, is presented in Table 2. Despite minor differences in wording and response options, the questions were comparable across timepoints. Table 3 provides the corresponding ALSPAC variable names and source file locations. All questionnaires and clinic assessments used in ALSPAC are publicly available to view on the study website. The specific questionnaires used in this analysis have also been uploaded to our project’s GitHub repository [ https://github.com/RochelleKnight/Estimating-age-of-menopause-in-mothers-in-the-ALSPAC-Study ].

**Table 1.** Description of source questionnaires and clinics.

Questionnaire/Clinic	Number of response/clinic attendees	Year of completion	Mean age (SD) at completion, years
Focus on Mothers 1 (FoM1) Clinic	4,822	2008–2011	47.40 (4.52)
Questionnaire T	4,144	2010–2012	48.58 (4.48)
Questionnaire U	4,423	2011–2012	49.65 (4.52)
Focus on Mothers 2 (FoM2) Clinic	2,889	2011–2013	50.32 (4.42)
Questionnaire V	4,650	2013–2014	51.27 (4.48)
Focus on Mothers 3 (FoM3) Clinic	3,003	2013–2014	51.56 (4.46)
Focus on Mothers 4 (FoM4) Clinic	2,905	2014–2015	52.63 (4.40)
Questionnaire Y	4,662	2020	57.66 (4.47)
Questionnaire MB *	4,569	2023	60.70 (4.43)

* Questionnaire MB followed a different structure for the reproductive health questions than the other questionnaires/clinics. It was therefore not used to assign dates of LMP as described under the section “ *Assigning date of LMP when self-reported date of LMP has been reported (completely or incompletely)”* however is included in the above table for completeness. See Extended Material for details on how data from Questionnaire MB were incorporated.

**Table 2.** Questions and response options in ALSPAC questionnaires and clinics used to determine age at menopause variable.

Question	Response								
	Questionnaire T	Questionnaire U	Questionnaire V	Questionnaire Y	Questionnaire MB	FoM1 clinic	FoM2 clinic	FoM3 clinic	FoM4 clinic
When was your last period?	Month, Year (or if month and year cannot be remembered age at the time could be reported)	Day, Month, Year	Month, Year (or if month and year cannot be remembered age at the time could be reported)	Month, Year (or if month and year cannot be remembered age at the time could be reported)	N/A	Day, Month, Year	Day, Month, Year (Date of last period was initially recorded as a complete date, with separate day, month and year variable extracted later. However, some women could only recall year of their last periods, hence a separate ‘year of last period’ variable was also available. This was used in cases where a date of last period was not provided.)	Day, Month, Year (Participants were also asked in a separate question the year their periods stopped which was used in cases where a date of last period was not provided.)	Day, Month, Year (Participants were also asked in a separate question the year their periods stopped which was used in cases where a date of last period was not provided.)
In the last 3 months have you had a period or menstrual bleeding?	Yes/No	N/A	Yes/No	Yes/No	Yes/No	Yes/No	Yes/No	Yes/No	Yes/No
In the last 12 months have you had a period or menstrual bleeding?	Yes/No	N/A	Yes/No	Yes/No	Yes/No	Yes/No	Yes/No	Yes/No	Yes/No
Were your periods stopped by: ^†^	Surgery; Chemotherapy or radiation therapy; Pregnancy or breast feeding; No obvious reason/menopause; Other reason *	N/A	Surgery; Chemotherapy or radiation therapy; Pregnancy or breast feeding; No obvious reason/menopause; Contraception *	Surgery; Chemotherapy or radiation therapy; Pregnancy or breast feeding; Menopause; Contraception; Other reason *	Hysterectomy; Pregnancy; Breastfeeding; Hormonal Coil; Menopause; Other reason; Unknown *	Hysterectomy; Ablation/resection; Oophorectomy; Chemotherapy or radiation therapy; Pregnancy or breast feeding; Menopause; Contraceptive: Coil; Contraceptive: Injection; Contraceptive: Implant; Contraceptive: Pill; Contraceptive: Other; Other medical reason; Other	Hysterectomy; Chemotherapy or radiation therapy; Pregnancy or breast feeding; Menopause; Other	Hysterectomy; Chemotherapy or radiation therapy; Pregnancy or breast feeding; Menopause; Hormonal coil; Other	Hysterectomy; Chemotherapy or radiation therapy; Pregnancy or breast feeding; Menopause; Hormonal coil; Other
Have you ever had any of the following operations? a)Removal of uterus (womb) and both ovaries (hysterectomy and bilateral oophorectomy) b)Removal of uterus (womb) only (hysterectomy) c)Removal of uterus (womb) and one ovary (hysterectomy and oophorectomy) d)Removal of both ovaries only (bilateral oophorectomy) e)Removal of one ovary only (oophorectomy)	Yes/No; If yes at what date (Month/Year). If date could not be remembered, age at operation could be reported	N/A	Yes/No; If yes at what date (Month/Year). If date could not be remembered, age at operation could be reported	Yes/No; If yes at what date (Month/Year). If date could not be remembered, age at operation could be reported	N/A	N/A	N/A	N/A	N/A
What forms of contraception are you using now? (Mark all that you have used in the past 3 months)	Withdrawal; The pill; IUCD/coil; Condom/sheath; Calendar/rhythm method; Diaphragm/cap; Spermicide; I have been sterilised; My partner has been sterilised; I am no longer fertile; None; Other	N/A	Withdrawal; The pill; Intrauterine device (coil, no hormones); Intrauterine device (coil, with hormones, such as a Mirena coil); Condom/sheath; Calendar/rhythm method; Diaphragm/cap; Spermicide; Contraceptive injection (such as Depo-Provera); Contraceptive implant (such as Implanon); I have been sterilised; My partner has been sterilised; I am no longer fertile; None; Other	Withdrawal; The pill; Intrauterine device (coil, no hormones); Intrauterine device (coil, with hormones, such as a Mirena coil); Condom/sheath; Calendar/rhythm method; Diaphragm/cap; Spermicide; Contraceptive injection (such as Depo-Provera); Contraceptive implant (such as Implanon); I have been sterilised; My partner has been sterilised; I am no longer fertile; None; Other	N/A	N/A	N/A	N/A	N/A
Currently taking oral contraceptives	N/A	Yes/No	N/A	N/A	N/A	Yes/No	Yes/No	Yes/No	Yes/No
Currently using contraceptive injection	N/A	Yes/No	N/A	N/A	N/A	Yes/No	Yes/No **	Yes/No	Yes/No
Currently using contraceptive implant	N/A	Yes/No	N/A	N/A	N/A	N/A	N/A	N/A	N/A
Currently using hormonal coil	N/A	Yes/No	N/A	N/A	N/A	N/A	Yes/No	Yes/No	Yes/No
Currently using contraceptive patch	N/A	Yes/No	N/A	N/A	N/A	N/A	N/A	N/A	N/A
Are you currently taking HRT?	Yes/No	N/A	Yes/No	Yes/No	Yes/No ***	Yes/No	Yes/No ***	Yes/No ***	Yes/No ***
At what age did you reach the menopause?	N/A	Years	N/A	N/A	N/A	N/A	N/A	N/A	N/A
Which of the following statements best describes your current menopause status?	N/A	N/A	N/A	N/A	I have been through the menopause; I am going through the menopause now; I have not yet started going through the menopause; I am not sure; I prefer not to answer	N/A	N/A	N/A	N/A
On what date did you complete this questionnaire?	Month, Year (whilst day could be reported, this was not included in the released data set)	Month, Year (whilst day could be reported, this was not included in the released data set)	Month, Year (whilst day could be reported, this was not included in the released data set)	Month, Year (whilst day could be reported, this was not included in the released data set)	Month, Year (whilst day could be reported, this was not included in the released data set)	Month, Year	Month, Year	Month, Year	Month, Year
Age at attendance	Years	Years	Years	Years	Years	Years	Years	Years	Years

^†^ If a participant answered ‘No’ to ‘In the last 12 months have you had a period or menstrual bleeding?’, they were then asked the question ‘Were your periods stopped by:’

* More than one response was allowed to be marked.

** Questioned asked as ‘Currently using contraceptive injection or implant’.

*** Split into three questions each asking about tablets, patches or creams.

**Table 3.** ALSPAC source and variable names.

Question	Source	Source file in ALSPAC	List of variable names in ALSPAC
When was your last period?	Questionnaire T	t_2a	t4811, t4812,t4813
	Questionnaire U	u_1a	U1040,U1041,U1042
	Questionnaire V	v_2a	V4811,V4812,V4813
	Questionnaire Y	Y_2a	Y5101, Y5102,Y5103
	Questionnaire MB	MB_1b	N/A
	FoM1	FOM1_3c	fm1ob123, fm1ob124, fm1ob125
	FoM2	FOM2_5b	fm2ob123, fm2ob124, fm2ob124a, fm2ob125
	FoM3	FOM3_3a	fm3ob123, fm3ob124, fm3ob124a, fm3ob125
	FoM4	FOM4_4a	fm4ob123, fm4ob124, fm4ob124a, fm4ob125
In the last 12 months have you had a period or menstrual bleeding?	Questionnaire T	t_2a	t4800
	Questionnaire U	u_1a	N/A
	Questionnaire V	v_2a	V4800
	Questionnaire Y	Y_2a	Y5070
	Questionnaire MB	MB_1b	MB4600
	FoM1	FOM1_3c	fm1ob120
	FoM2	FOM2_5b	fm2ob120
	FoM3	FOM3_3a	fm3ob120
	FoM4	FOM4_4a	fm4ob120
In the last 3 months have you had a period or menstrual bleeding?	Questionnaire T	t_2a	t4810
	Questionnaire U	u_1a	N/A
	Questionnaire V	v_2a	V4810
	Questionnaire Y	Y_2a	Y5100
	Questionnaire MB	MB_1b	MB4600
	FoM1	FOM1_3c	fm1ob126
	FoM2	FOM2_5b	fm2ob126
	FoM3	FOM3_3a	fm3ob126
	FoM4	FOM4_4a	fm4ob126
What were your periods stopped by?	Questionnaire T	t_2a	t4801, t4802, t4803, t4804, t4805
	Questionnaire U	u_1a	N/A
	Questionnaire V	v_2a	V4801, V4802, V4803, V4804, V4805
	Questionnaire Y	Y_2a	Y5080, Y5081, Y5082, Y5083, Y5084, Y5085
	Questionnaire MB	MB_1b	MB4610, MB4620, MB4630, MB4640, MB4650, MB4660, MB4670
	FoM1	FOM1_3c	fm1ob121
	FoM2	FOM2_5b	fm2ob121
	FoM3	FOM3_3a	fm3ob121
	FoM4	FOM4_4a	fm4ob121
Have you ever had any of the following operations? **a)** Removal of uterus (womb) and both ovaries (hysterectomy and bilateral oophorectomy) **b)** Removal of uterus (womb) only (hysterectomy) **c)** Removal of uterus (womb) and one ovary (hysterectomy and oophorectomy) **d)** Removal of both ovaries only (bilateral oophorectomy) **e)** Removal of one ovary only (oophorectomy)	Questionnaire T	t_2a	t4700, t4701, t4702, t4703, t4710, t4711, t4712, t4713, t4720, t4721, t4722, t4723, t4730, t4731, t4732, t4733, t4740, t4741, t4742, t4743
	Questionnaire U	u_1a	N/A
	Questionnaire V	v_2a	V4700, V4701, V4702, V4703, V4710, V4711, V4712, V4713,V4720, V4721, V4722, V4723, V4730, V4731, V4732, V4733, V4740, V4741, V4742, V4743
	Questionnaire Y	Y_2a	Y5020, Y5021, Y5022, Y5023, Y5030, Y5031, Y5032, Y5033, Y5040, Y5041, Y5042, Y5043, Y5050, Y5051, Y5052, Y5053, Y5060, Y5061, Y5062, Y5063
	Questionnaire MB	MB_1b	N/A
	FoM1	FOM1_3c	N/A
	FoM2	FOM2_5b	N/A
	FoM3	FOM3_3a	N/A
	FoM4	FOM4_4a	N/A
What forms of contraception are you using now? (Mark all that you have used in the past 3 months)	Questionnaire T	t_2a	t4521, t4522
	Questionnaire U	u_1a	N/A
	Questionnaire V	v_2a	V4551, V4552, V4553, V4559, V4560
	Questionnaire Y	Y_2a	Y5001, Y5002, Y5003, Y5008, Y5009
	Questionnaire MB	MB_1b	N/A
	FoM1	FOM1_3c	N/A
	FoM2	FOM2_5b	N/A
	FoM3	FOM3_3a	N/A
	FoM4	FOM4_4a	N/A
Currently taking oral contraceptives	Questionnaire T	t_2a	N/A
	Questionnaire U	u_1a	U1030
	Questionnaire V	v_2a	N/A
	Questionnaire Y	Y_2a	N/A
	Questionnaire MB	MB_1b	N/A
	FoM1	FOM1_3c	fm1ob100
	FoM2	FOM2_5b	fm2ob100
	FoM3	FOM3_3a	fm3ob100
	FoM4	FOM4_4a	fm4ob100
Currently using contraceptive injection	Questionnaire T	t_2a	N/A
	Questionnaire U	u_1a	U1031
	Questionnaire V	v_2a	N/A
	Questionnaire Y	Y_2a	N/A
	Questionnaire MB	MB_1b	N/A
	FoM1	FOM1_3c	fm1ob101
	FoM2	FOM2_5b	fm2ob101
	FoM3	FOM3_3a	fm3ob101
	FoM4	FOM4_4a	fm4ob101
Currently using contraceptive implant	Questionnaire T	t_2a	N/A
	Questionnaire U	u_1a	U1032
	Questionnaire V	v_2a	N/A
	Questionnaire Y	Y_2a	N/A
	Questionnaire MB	MB_1b	N/A
	FoM1	FOM1_3c	N/A
	FoM2	FOM2_5b	N/A
	FoM3	FOM3_3a	N/A
	FoM4	FOM4_4a	N/A
Currently using hormonal coil	Questionnaire T	t_2a	N/A
	Questionnaire U	u_1a	U1033
	Questionnaire V	v_2a	N/A
	Questionnaire Y	Y_2a	N/A
	Questionnaire MB	MB_1b	N/A
	FoM1	FOM1_3c	N/A
	FoM2	FOM2_5b	fm2ob102
	FoM3	FOM3_3a	fm3ob102
	FoM4	FOM4_4a	fm4ob102
Currently using contraceptive patch	Questionnaire T	t_2a	N/A
	Questionnaire U	u_1a	U1034
	Questionnaire V	v_2a	N/A
	Questionnaire Y	Y_2a	N/A
	Questionnaire MB	MB_1b	N/A
	FoM1	FOM1_3c	N/A
	FoM2	FOM2_5b	N/A
	FoM3	FOM3_3a	N/A
	FoM4	FOM4_4a	N/A
Are you currently taking HRT?	Questionnaire T	t_2a	t4961
	Questionnaire U	u_1a	N/A
	Questionnaire V	v_2a	V4955
	Questionnaire Y	Y_2a	Y5140
	Questionnaire MB	MB_1b	MB4790, MB4800, MB4810, MB4810
	FoM1	FOM1_3c	fm1ob110
	FoM2	FOM2_5b	fm2ob110a, fm2ob110b, fm2ob110c
	FoM3	FOM3_3a	fm3ob110a, fm3ob110b, fm3ob110c
	FoM4	FOM4_4a	fm4ob110a, fm4ob110b, fm4ob110c
On what date did you complete this questionnaire/attend this clinic?	Questionnaire T	t_2a	t9990a, t9990b
	Questionnaire U	u_1a	U2010, U2011
	Questionnaire V	v_2a	V9990A, V9990B
	Questionnaire Y	Y_2a	Y9990, Y9991
	Questionnaire MB	MB_1b	MB9021, MB9022
	FoM1	FOM1_3c	fm1a010a, fm1a010b
	FoM2	FOM2_5b	fm2a010a, fm2a010b
	FoM3	FOM3_3a	fm3a010a, fm3a010b
	FoM4	FOM4_4a	fm4a010a, fm4a010b
Age at completion of questionnaire/attendance of clinic	Questionnaire T	t_2a	t9994
	Questionnaire U	u_1a	U2021
	Questionnaire V	v_2a	V9996
	Questionnaire Y	Y_2a	Y9992
	Questionnaire MB	MB_1b	MB9510
	FoM1	FOM1_3c	fm1a011
	FoM2	FOM2_5b	fm2a011
	FoM3	FOM3_3a	fm3a011
	FoM4	FOM4_4a	fm4a011
Age at menopause	Questionnaire U	u_1a	U1021
Which of the following statements best describes your current menopause status?	Questionnaire MB	MB_1b	MB4680

In brief, information was obtained on the day, month and year of reported date of LMP, whether a period occurred in the last 12 months (yes/no), whether a period occurred in the last 3 months (yes/no), what caused menstruation to stop if relevant, occurrence of any surgical operations relating to reproductive organs, and if the women were taking hormonal contraceptives or HRT.

## Estimating the date of last menstrual period

We estimated the date of LMP for each woman at each of the eight timepoints. For each woman, we ordered response timepoints by the time of questionnaire completion/clinic attendance. ‘ **Year of attendance**’ refers to the year they responded to the questionnaire/year of clinic attendance, and ‘ **date of attendance**’ refers to the date they responded to the questionnaire/date of clinic attendance. To ensure participant confidentiality, only the month and year of date of attendance are available to researchers. Therefore, we set the day to the midpoint of the month (the 15 ^th^). We prioritised the clinic data as the primary source of data for women who completed a questionnaire and attended a clinic assessment in the same month. To estimate the date of women’s LMP, we combined three key variables which were asked as separate questions at all timepoints:

1. **Self-reported date of LMP**: date of LMP (reported as dd/mm/yyyy). Some women reported the complete date, some reported month and year, and some reported only year.
2. **Whether they had a period or menstrual bleeding in the last 3 months**: yes/no response indicating the presence versus absence of a period or menstrual bleeding in the last 3 months.
3. **Whether they had a period or menstrual bleeding in the last 12 months**: yes/no response indicating the presence versus absence of a period or menstrual bleeding in the last 12 months.

Before estimating the LMP, we sense-checked the 3- and 12-month bleeding history questions to ensure internal consistency (see Extended Material Table 1 for details on recoding rules and inconsistencies resolved).

**Figure 1.**
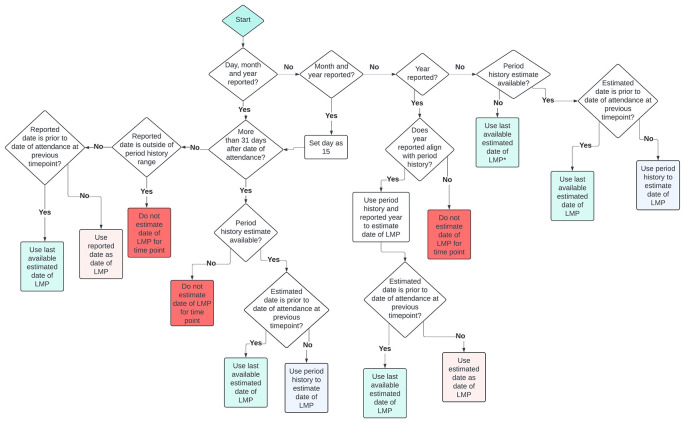
Flowchart of method used to assign date of last menstrual period. *subject to no self-reported LMP date at a given timepoint, the woman reported no menstrual periods in the last 12 months, and the previous timepoint occurred within the last 2 years. Else no date of LMP estimated for timepoint. Period history estimate derived from questions “In the last 12 months have you had a period or menstrual bleeding?” and “In the last 3 months have you had a period or menstrual bleeding?”

A flow chart of the approach used to estimate date of LMP at each timepoint is presented in Figure 1, and the approach, including illustrative case examples, is detailed below. In brief, LMP dates were estimated based on the available self-reported date of LMP and/or bleeding history (period in the last 12 and/or 3 months), with consistency checks applied to ensure plausible alignment across variables.

### Assigning date of LMP when self-reported date of LMP has been reported (completely or incompletely)

When asked to report their date of LMP, some women reported a complete date (dd/mm/yyyy), whilst others reported month and year, or only the year of their LMP.

***1. Complete date of LMP has been reported***

When a complete date of LMP was reported, we assigned this date as the date of LMP.

***2. Both month and year of LMP were reported, but day is missing***

When both month and year of LMP were reported, but the day was missing, we set the day to the midpoint of the month (the 15 ^th^) and used this to assign the date of LMP.

***3. Only year of LMP was reported, and month and day were missing***

When only the year of LMP was reported, we combined information regarding reported period or menstrual bleeding in the last 3 and 12 months to assign the date of LMP. We did that as follows:

**Report of no period or menstrual bleeding in the last 3 and 12 months**

**Figure 2.**
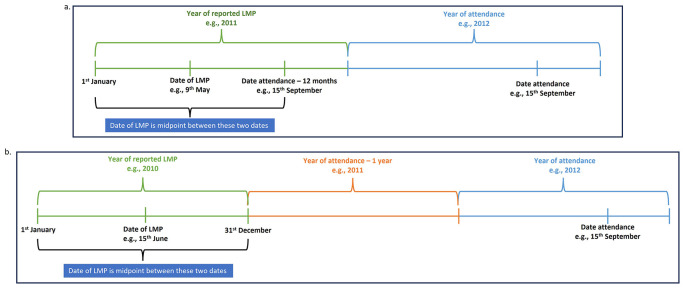
Assigning date of LMP when only year of LMP was reported and report of no period or menstrual bleeding in the last 3 and 12 months examples.

a) When the reported year of LMP matched the year of attendance, we did not assign a date of LMP, as this was inconsistent with no period or menstrual bleeding in the last 12 months. *For example, if the date of attendance was 15 ^th^ September 2012, and the reported year of LMP was 2012, this would be inconsistent with no period or menstrual bleeding in the last 12 months.*
b) When the reported year of LMP was the year before the year of attendance, we assigned the date of LMP as the midpoint between the 1 ^st^ January of the reported LMP year and the date one year prior to date of attendance ( Figure 2a). *For example, if the date of attendance was 15 ^th^ September 2012, and the reported year of LMP was 2011, the date of LMP was assigned as the midpoint between 1 ^st^ January 2011 and 15 ^th^ September 2011.*
c) When the reported year of LMP was more than one year before year of attendance, we assigned the date of LMP as 15 ^th^ June of the reported LMP year Figure 2b). *For example, if the date of attendance was 15 ^th^ September 2012, and the reported year of LMP was 2010, the date of LMP was assigned as the 15 ^th^ June 2010.*

**Report of period or menstrual bleeding in the last 12 months but not in the last 3 months**

**Figure 3.**
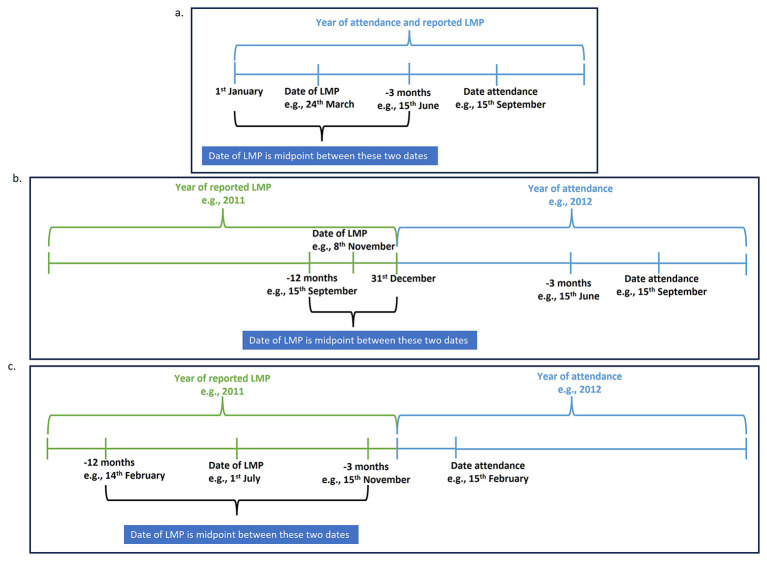
Assigning date of LMP when only year of LMP was reported and report of period or menstrual bleeding in the last 12 months but not in the last 3 months examples.

a) When the reported year of LMP matched the year of attendance, and the 3-month window prior to attendance fell within the same year, we assigned the date of LMP as the midpoint between 1 ^st^ January of the reported LMP year and the date three months before the date of attendance ( Figure 3a). *For example, if the date of attendance was 15 ^th^ September 2012, and the reported year of LMP was 2012, the date of LMP was assigned as the midpoint between 1 ^st^ January 2012 and 15 ^th^ June 2012.*
b) When the reported year of LMP was the year before the year of attendance and the 3-month window prior to the date of attendance did not extend into the reported LMP year, we assigned the LMP date as the midpoint between one year before the date of attendance and 31 ^st^ December of the reported LMP year ( Figure 3b). *For example, if the date of attendance was 15 ^th^ September 2012, and the reported year of LMP was 2011, the date of LMP was assigned as the midpoint between 15 ^th^ September 2011 and 31 ^st^ December 2011.*
c) When the reported year of LMP was the year before the year of attendance, and the 3-month window prior to date of attendance extended into the reported LMP year, we assigned the LMP date as the midpoint between one year before date of attendance and three months before date of attendance ( Figure 3c). *For example, if the date of attendance was 15 ^th^ February 2012, and the reported year of LMP was 2011, the date of LMP was assigned as the midpoint between 14 ^th^ February 2011 and 15 ^th^ November 2011.*
d) We did not assign a date of LMP when the reported year of LMP matched the year of attendance, but the 3-month window prior to date of attendance extended into the previous year. This is because it would then be inconsistent with no menstrual bleeding in the last 3 months. *For example, if the date of attendance was 15 ^th^ February 2012, and the reported year of LMP was 2012, a period lasting longer than 3 months but less than 12 months would fall in 2011, not 2012.*
e) We did not assign a date of LMP when the self-reported year of LMP was more than one year before the year of attendance, as this would be inconsistent with having a period or menstrual bleeding in the last 12 months. *For example, if the date of attendance was 15 ^th^ February 2012 and the reported year of LMP was 2010 or earlier, any date within that year would fall outside the 12-month window.*

**Report of period or menstrual bleeding in the last 3 months**

**Figure 4.**
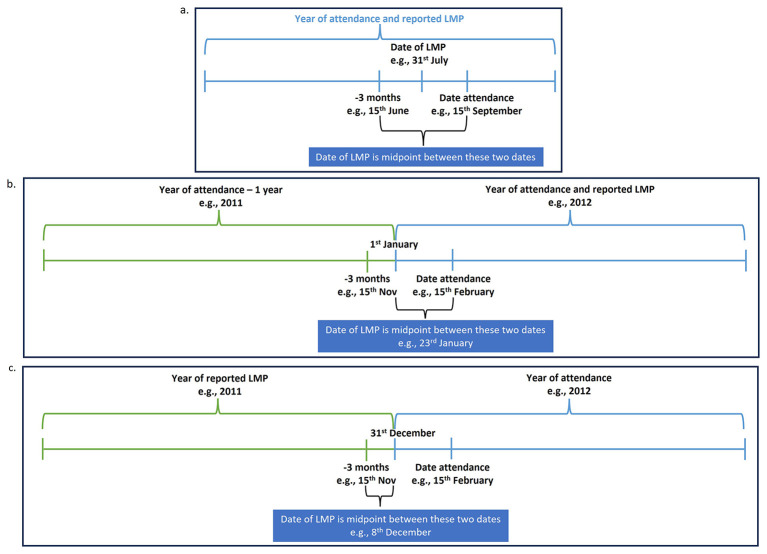
Assigning date of LMP when only year of LMP was reported and report of period or menstrual bleeding in the last 3 and 12 months.

a) When the reported year of LMP matched the year of attendance and the 3-month window prior to the date of attendance did not extend into the previous year, we assigned the LMP date as the midpoint between the date of attendance and the date three months earlier ( Figure 4a). *For example, if the date of attendance was 15 ^th^ September 2012, and the reported year of LMP was 2012, the date of LMP was assigned as the midpoint between 15 ^th^ June 2012 and 15 ^th^ September 2012.*
b) When the reported year of LMP matched the year of attendance and the 3-month window prior to the date of attendance extended into the previous year, we assigned the LMP date as the midpoint between 1 ^st^ January of the reported LMP year and the date of attendance ( Figure 4b). *For example, if the date of attendance was 15 ^th^ February 2012, and the reported year of LMP was 2012, the date of LMP was assigned as the midpoint between 1 ^st^ January 2012 and 15 ^th^ February 2012.*
c) When the reported year of LMP was the year before the year of attendance and the 3-month window prior to the date of attendance extended into the reported LMP year, we assigned the date of LMP as the midpoint between three months before the date of attendance and 31 ^st^ December of the reported LMP year ( Figure 4c). *For example, if the date of attendance was 15 ^th^ February 2012, and the reported year of LMP was 2011, the date of LMP was assigned as the midpoint between 15 ^th^ November 2011 and 31 ^st^ December 2011.*
d) For all other scenarios, we did not assign a date of LMP.

### Consistency checks

*a.*
***Assigned date of LMP is consistent with reported menstruation in the previous 3 and 12 months***

For women with an assigned LMP date (as described in sections “1. Complete date of LMP has been reported” or “2. Both month and year of LMP were reported, but day is missing”), we checked consistency with responses to whether they had a period or menstrual bleeding in the last 3 and 12 months. To minimise data loss while maintaining plausibility, we applied flexible thresholds based on these responses.

**Figure 5.**
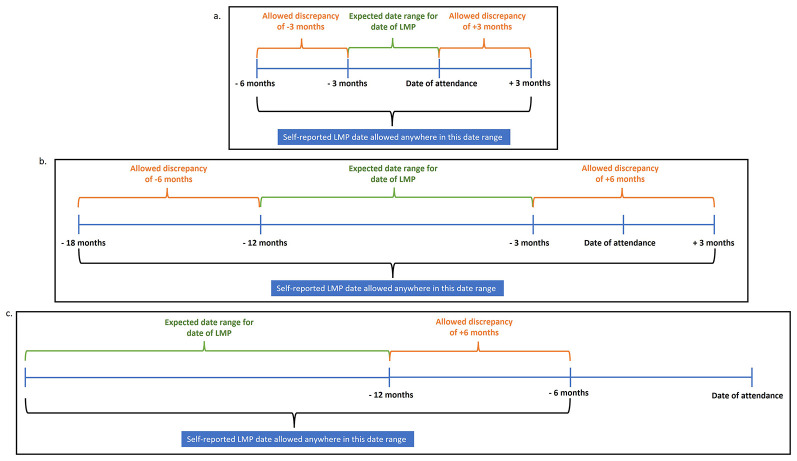
Consistency check: Assigned date of LMP is consistent with reported menstruation in the previous 3 and 12 months examples.

• **Reported they had menstruated in the last 3 months:** We assumed LMP should fall within this period but allowed a ± 3-month buffer to account for reporting error. Therefore, assigned dates were accepted if they fell from 3 months before the start of that period to 3 months after the date of attendance ( Figure 5a).
• **Reported they had menstruated in the last 12 months but not in the last 3 months:** A wider ±6-month buffer was applied around the expected LMP window ( Figure 5b).
• **Reported they had not menstruated in the last 12 months:** We assumed the LMP occurred at least 12 months prior to the date of attendance but allowed a 6-month buffer. Assigned LMP dates therefore had to be at least 6 months before the date of attendance ( Figure 5c).

Assigned LMP dates falling outside these allowable windows were set to missing. Assigned dates of LMP that occurred after the date of attendance (i.e., future dates) were subject to additional checks (see below).

*b.*
***Assigned date of LMP after the date of attendance (i.e., date in the future)***

Since the exact date of clinic attendance or questionnaire completion was not available to researchers, all attendance dates were standardised to the 15 ^th^ of the month. This standardisation could result in instances where the assigned LMP date appeared to fall after the date of attendance. For example, if the actual attendance occurred on 31 ^st^ May and the true LMP was also on 31 ^st^ May, but the attendance date was standardised to 15 ^th^ May, a discrepancy of 16 days would arise. To address this and minimize data loss, we allowed the assigned LMP date to occur up to 31 days after the standardised attendance date, accounting for potential misreporting of the month. If the assigned date of LMP exceeded 31 days beyond the attendance date, it was set to missing.

*c.*
***Assigned date of LMP is prior to date of attendance at the previous timepoint***

If the assigned date of LMP for a given timepoint occurred before the date of attendance at the previous timepoint, it was set to missing, unless no LMP date had been assigned for the previous timepoint. This approach assumes that if an LMP date falls before the date of attendance of an earlier timepoint, it is more likely to be influenced by recall error than the estimate provided at the previous timepoint. Therefore, we retained the LMP date that was most proximal to the reporting period.

*For example, consider a woman who attended her first timepoint on 15 ^th^ May 2013 and reported her LMP as 17 ^th^ June 2008. At her second timepoint on 15 ^th^ October 2015, she reported her LMP as 31 ^st^ February 2009. Although the second timepoint’s LMP date is more recent, it predates the attendance date of the first timepoint and therefore the LMP date reported in the second timepoint was set to missing. However, if no LMP date had been assigned at the first timepoint, the LMP date from the second timepoint would be retained.*

### Assigning date of LMP when self-reported date of LMP has not been provided

In cases where no self-reported date of LMP was available, we relied on information about reported menstrual bleeding in the last 3 and 12 months, as outlined below.

***4. Assigning date of LMP using responses relating to menstruation in the last 3 and 12 months***

We assigned the date of LMP using reported period or menstrual bleeding in the last 3 and 12 months in the following two scenarios:

i. **No information on self-reported date of LMP:** The woman did not report any part of the LMP date (day, month or year).
ii. **Invalid self-reported date of LMP:** The self-reported LMP date was more than 31 days after the date of attendance.

As previously explained, any LMP date reported as more than 31 days after the date of attendance was set to missing. However, to minimise the number of timepoints with missing LMP dates, we assumed that a future-dated LMP, although misreported, still likely indicated that the woman was menstruating. Therefore, in both scenarios, we assigned the LMP date using responses relating to menstruation in the last 3 and 12 months, as follows:

*a)*
***Reported having a menstrual period in the last 3 months***

If the woman reported menstruation in the last 3 months, the LMP date was assigned as the midpoint between the date of attendance and 3 months prior (calculated as 45 days prior to the date of attendance).

*b)*
***Reported having a menstrual period in the last 12 months but not in the last 3 months***

If the woman reported menstruation within the last 12 months but not in the last 3 months, the LMP date was assigned as the midpoint between 12 months before and 3 months before the date of attendance (calculated as 228 days prior to date of attendance).

*c)*
***Reported having no menstrual period in the last 12 months***

If the woman reported no menstruation in the last 12 months, an LMP date was unable to be assigned and was set as missing.

### Consistency check

*a.*
***Assigned date of LMP is prior to date of attendance at the previous timepoint***

The same consistency check as described above under the section **‘Estimating date of LMP when self-reported date of LMP has been reported (completely or incompletely)’** was also performed.

### Assigning date of last menstrual period using estimated date from the previous timepoint

If an LMP date could not be assigned using self-reported LMP date or menstrual bleeding history (i.e., reports of menstrual bleeding in the last 3 or 12 months combined with the date of attendance), we used the most recent LMP from a previous timepoint to minimise missing data.

Specifically, we carried forward the most recent LMP from an earlier timepoint in the following scenarios:

i. The assigned LMP date at a given timepoint fell before the date of attendance of the previous timepoint.
ii. There was no self-reported LMP date at a given timepoint, the woman reported no menstrual periods in the last 12 months, and the previous timepoint occurred within the last 3 years.

In all other cases, the LMP date remained as missing.

The rationale for the second scenario was to avoid misclassifying menopausal status by inferring menopause too early. Carrying forward an LMP from an earlier timepoint could misleadingly suggest a woman had reached menopause because more than 12 months had passed since her previous timepoint (and hence more than 12 months since her last recorded period). Given the variability in time gaps between timepoints—sometimes up to 10 years—such an approach could result in significant errors in estimating age at menopause.

For example, consider a woman who attended a timepoint on 15 ^th^ October 2010 and reported an LMP of 17 ^th^ September 2010. She next attended on 15 ^th^ February 2015, did not report an LMP date, but stated that she had not had a period in the last 12 months. If we assigned her 2010 LMP to this later timepoint it would imply she was postmenopausal, even though she may have continued menstruating after 2010 – information not captured due to the long gap between assessments. To address this, we only carried forward LMP dates if the previous timepoint occurred within three years of the current one, limiting the potential error. For women who reported no periods in the last 12 months and had an LMP up to three years prior, this implies that menopause likely occurred within the preceding two years — such as between ages 51 and 53 for a woman attending at age 54. We considered this level of uncertainty acceptable for classifying menopausal status, given the limitations of self-reported data. See Extended Material for details on how data from Questionnaire MB were incorporated.

## Estimating age at menopause

Age at natural menopause was determined using both a derived measure based on menstrual bleeding history data and self-reported age at menopause, whenever available.

Having assigned LMP dates as described above, we defined the final menstrual period (menopause) as occurring if a woman’s most recent LMP was more than 365 days before the date of attendance at her last assessment. Age at menopause was then defined as age at FMP. Self-reported age at menopause was derived from multiple questionnaires. Questionnaires T, V, and Y asked, *“What was your age at your last menstrual period?”.* If the reported age was at least one year prior to the respondent’s current age, it was assigned as self-reported age at menopause. Questionnaire U (completed in 2011–2012 by 4,423 women) directly asked, *“What was your age at menopause?”.* The maximum self-reported age at menopause across Questionnaires U, T, V, and Y was assigned as each woman’s self-reported age at menopause.

To ensure consistency between sources, we applied a validity check: self-reported age at menopause had to be no more than two years earlier than the age at the most recent LMP, otherwise it was excluded. This helped reduce instances where self-report and LMP data conflicted in implausible ways. For example, consider a woman who reported a LMP within the last 12 months at age 52, meaning she was not yet classified as postmenopausal. At a later timepoint, aged 54, she reported her last menstrual period occurred at age 50 via the question *“What was your age at your last menstrual period?”.* If taken at face value, this would suggest she was already postmenopausal by age 52, contradicting her earlier LMP report. To allow for some inconsistency in recall or reporting, we accepted self-reported ages at menopause only if they were after the most recent LMP, or within two years prior to it. This threshold provided a balance between data inclusion and plausibility.

If a woman only had self-reported age at menopause, this value was used. Otherwise, we prioritised the menopause age derived from longitudinal menstrual reports, as it is less likely to be affected by recall bias.

## Exclusions

As age of menopause is estimated based on menstrual bleeding patterns, we also considered factors that affect menstrual bleeding and therefore could result in an inaccurate assessment of menopausal status. These include surgery of reproductive organs, use of contraceptives and HRT, and other reasons such as chemotherapy or radiation therapy, ablation/resection, pregnancy, or breastfeeding.

### Surgical reasons

All women were asked in questionnaires and clinics whether they had undergone any surgeries to remove their reproductive organs (see Table 2 for full list of options). Response options that would result in the cessation of menstruation were as follows:

• Hysterectomy with bilateral oophorectomy (removal of uterus and both ovaries)
• Bilateral oophorectomy (removal of both ovaries)
• Hysterectomy (removal of uterus)
• Hysterectomy with unilateral oophorectomy (removal of uterus and one ovary)

Women who reported any of these surgeries were asked to provide the date and age at which the procedure was performed. If a woman did not explicitly report undergoing surgery but provided either an age or a date of surgery, it was assumed that she had the procedure. Conversely, women who reported surgery but did not provide a date or age were excluded.

As a separate question, women who reported no menstrual bleeding in the last 12 months were asked to indicate the reason for cessation (see Table 2 for full list of options). Surgical reasons included: Surgery, Hysterectomy and Oophorectomy, without further detail. We assumed that reports of ‘Surgery’ and ‘Oophorectomy’ would result in the cessation of all subsequent menstruation. The women were not asked to report the date of surgery, hence the date and age at questionnaire completion or clinic attendance were used as proxies.

The overall date and age at surgery were taken as the earliest reported values. Any timepoints following surgery were excluded. Estimations of date of LMP were made for timepoints preceding surgery. If self-reported age at menopause age derived from Questionnaires T, U, V and Y was greater than or equal to the surgery age, it was set to missing.

### Contraception and HRT

At all eight timepoints, women were asked whether they were currently using hormonal contraceptives or HRT. Table 2 summarises the specific questions and response options related to contraceptive and HRT use for all for all questionnaires and clinic assessments. Because hormonal medications can affect menstrual bleeding patterns, menopausal status cannot be determined when these medications are being used. Therefore, any timepoints at which current use of hormonal contraceptives or HRT was reported were excluded.

### Periods stopped due to other reasons

Women who reported no menstrual bleeding in the last 12 months were asked to indicate the reason for cessation (see Table 2 for full list of options). Timepoints were excluded if the reported cause was chemotherapy or radiation therapy; ablation/resection; pregnancy, or breastfeeding; or other reason/other medical reason.

## Description of the population

**Figure 6.**
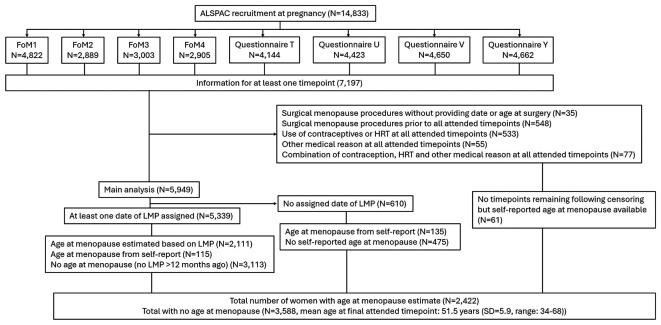
Flow of participants through the age at natural menopause derivation process.

Figure 6 shows participant flow from recruitment through to analysis. After accounting for withdrawal of consent, 14,833 unique G0 women were enrolled in the ALSPAC study, of whom 7,197 responded to at least one relevant timepoint. Thirty-five women were excluded due to reporting a surgical procedure that would have ceased menstruation without providing the date or age at surgery. After applying exclusion criteria, 5,949 women remained, contributing an average of 3.6 responses each. We were able to assign at least one LMP date for 5,339 of these women.

We derived an age at natural menopause - using both age derived from menstrual bleeding history and self-reported age - for 2,422 women. The overall average age at menopause was 49.4 years (SD = 4.1, range: 30–63 years, median = 50 years). Of these, 2,111 women had an age derived from menstrual history data (mean = 49.6 years, SD = 4.1, range: 30–63), and 311 based on self-reported age at menopause (mean = 48.2 years, SD = 4.0, range: 34–61). A total of 1,322 women had both a derived and self-reported age at menopause, with a correlation 0.75 between the two measures.

We were unable to derive an age at natural menopause for 3,588 women. At their final attended timepoint, these women had a mean age of 51.5 years (SD: 5.9, range: 34–68). Of them, 1,982 had experienced an LMP within the last 12 months. An additional 1,483 women (mean age = 56.9 years, SD = 3.1) reported that they had not had a period in the last 12 months, likely indicating they were postmenopause, however lacked sufficient data to estimate a final menstrual period.

**Figure 7.**
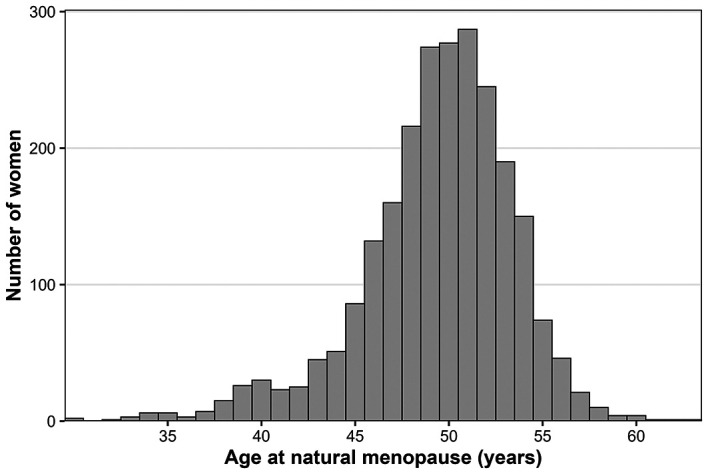
Histogram of age at natural menopause in the ALSPAC mothers cohort.

Figure 7 displays the distribution of age at menopause within the sample.

## In context with other studies

The UK Biobank asked women to report their age at menopause during the baseline assessment and the mean reported age was 49.8 years (median 50, SD = 5.1). The Study of Women’s Health Across the Nation (SWAN Study), a longitudinal study in the US, found a median age at natural menopause of 51.4 years in its cross-sectional screener data (n = 14,620) ^31^ and slightly higher at 52.5 years among longitudinal cohort participants (n = 1,483). ^32^ The higher estimate in the longitudinal cohort likely reflects selection factors: women who had already reached menopause before age 42 were excluded from follow-up, and attrition over time disproportionately affected women with characteristics linked to earlier menopause (e.g., smokers, lower education, poorer health). Other studies report a median self-reported age at menopause among White women from industrialized countries ranging between 50 and 52 years. ^33– 37^

In our sample, the mean age at menopause was 49.4 years (median 50 years), slightly lower than in other studies but still comparable. This discrepancy may be partly due to the mean age at several timepoints falling below the typical menopausal age range. As a result, women who experienced menopause earlier may have been more likely to be captured, while those who reached menopause at older ages may have been underrepresented. This could have biased the estimated distribution of menopausal age toward younger ages, potentially explaining the lower age at menopause in our sample compared to other studies. When further data is available from more recent follow-ups, this estimation should be re-calculated.

## Strengths and limitations of the data

One of the key strengths of the ALSPAC dataset is the availability of repeated data on menstrual cycles. This longitudinal information provides a detailed view of menstrual changes over time, capturing the often-irregular patterns characteristic of the menopause transition. As a result, it allows for more accurate estimation of age at menopause compared to relying solely on retrospective self-reports, which are prone to recall error. ^21, 22^ Additionally, the repeated data support future work to classify woman into menopausal stages - premenopause, perimenopause and postmenopause - using the Stages of Reproductive Aging Workshop (STRAW) + 10 ^38^ criteria.

A broad challenge was the potential for incorrect estimation of age at menopause due to missing, incomplete, or inconsistent reporting of menstrual history. Responses to “ *In the last 12 months have you had a period or menstrual bleeding?*”, “ *In the last 3 months have you had a period or menstrual bleeding?*” and “ *When was your last period?*” did not always align. Although consistency checks were performed to reconcile discrepancies, misreporting of LMP may still have affected the estimation of age at menopause.

In some cases, we identified likely misclassification. For example, 80 women reported a period of amenorrhea lasting more than 12 months at one timepoint, suggesting menopause, but subsequently reported menstruating within the last year at a later timepoint. This highlights the limitations of relying on self-reported data to determine menopausal status. Ultimately, we used the most recent plausible reported LMP to define age at menopause. In longitudinal data with repeated and occasionally conflicting reports, a single LMP must be selected to estimate age at menopause, and we considered the most recent plausible report to provide the most reliable representation of menstrual status across follow-up. As a result, 37 of these women do not have an estimated age at menopause, despite previously indicating a period of amenorrhea consistent with menopause.

Another limitation is the lack of information on when women discontinued hormonal contraceptives or HRT. While current use was recorded, there was no data on the timing of discontinuation. Because hormonal medications can obscure natural bleeding patterns, menopausal status—and particularly the timing of the final menstrual period—cannot be reliably determined while a woman is using them. We therefore excluded timepoints where hormone use was reported. However, if a woman reported hormone use at one timepoint but not at a later timepoint, we lacked information on how long had elapsed since discontinuation, and could not determine natural menstrual cycles had resumed. Consequently, a reported LMP could reflect bleeding prior to starting hormones, a withdrawal bleed, or a natural period. Some women may have also reached menopause while still using hormonal medications, meaning the menopausal transition was not captured, and their age at menopause could not be estimated. These limitations may have affected the accuracy of LMP reporting and, consequently, the estimation of menopausal age.

While the exclusion of timepoints where hormonal medications were reported was necessary, it may have introduced bias into the age at natural menopause. Overall, 35% of women ever reported the use of hormonal medications. Of these, 25% had a derived age at natural menopause, compared with 39% of women who never reported hormonal medication use. Women who used hormonal medications may therefore be underrepresented among those with a derived age at natural menopause. If women experiencing more severe menopausal symptoms were more likely to initiate HRT before their final menstrual period, this could lead to underrepresentation of women with more symptomatic menopausal transitions. Researchers using this variable should therefore consider the potential for selection bias and assess the impact of hormonal medication use where possible. The often large gaps between attended timepoints also posed challenges. While the average interval between attended timepoints was 2.1 years, some exceeded 10 years. In such cases, when the only available information was a report of no menstruation in the last 12 months, menopause likely occurred sometime during the intervening years. However, without more precise or frequent data, age at menopause could not be reliably assigned, contributing to potential misclassification and underestimation of menopausal age.

Finally, the structure of Questionnaire Y limited our ability to assign LMP dates at that timepoint. Whereas most questionnaires asked all women about menstruation in the last 12 and 3 months and to report the date of their LMP, Questionnaire Y only asked for an LMP date if the woman reported menstruating in the last 12 months. As a result, we could only assign an LMP date for a subset of women at this timepoint—those still menstruating—limiting the usefulness of these data for estimating age at menopause and adding to the broader challenges described above.

## Data availability

Access to ALSPAC data is available through a system of managed open access. Application steps to request access to ALSPAC data are highlighted below.

1. Please read the ALSPAC access policy (PDF, 621kB) which describes the process of accessing the data and samples in detail, and outlines the costs associated with doing so.
2. You may also find it useful to browse our fully searchable research proposals database, which lists all research projects that have been approved since April 2011.
3. Please submit your research proposal for consideration by the ALSPAC Executive Committee. You will receive a response within 10 working days to advise you whether your proposal has been approved.

Any questions regarding data or sample access should be directed to alspac-data@bristol.ac.uk (data) or bbl-info@bristol.ac.uk (samples).

The final derived ‘Age at Menopause’ variable will returned to ALSPAC and made available to other researchers as part of the ALSPAC data resource.

Extended Materials and source code are available on GitHub: https://github.com/RochelleKnight/Estimating-age-of-menopause-in-mothers-in-the-ALSPAC-Study

Archived software available from: https://doi.org/10.5281/zenodo.17236215

License: MIT License.

## Author contributions

**Rochelle Knight:** Conceptualization, Methodology, Software, Data Curation, Writing – Original Draft. **Abigail Fraser:** Conceptualization, Methodology, Writing – Review & Editing, Supervision. **Carol Joinson:** Conceptualization, Methodology, Writing – Review & Editing, Supervision. **Ana Gonçalves Soares:** Conceptualization, Methodology, Software, Writing – Review & Editing, Supervision.
