# Supplementary material for: Estimating age of menopause in mothers in the ALSPAC Study: A data note
Source: Wellcome Open Res. 2026 Jun 25;10:631. Originally published 2025 Nov 10. [Version 2] doi: 10.12688/wellcomeopenres.24760.2 (PMC12728343; doi:10.12688/wellcomeopenres.24760.2)
Supplement: Peer review history

# Reviewer report: Reviewer response for version 1

**DOI:** 10.21956/wellcomeopenres.27277.r139237

**Published:** 2025-12-27

**Authors:** Sostina Spiwe Matina

**Affiliations:** 1 Wits University, Johannesburg, Gauteng, South Africa

1. The Rationale needs to be more detailed.

 The paper clearly states what the authors want to achieve (a reproducible menopause variable for ALSPAC) and offers general justification (menopause timing is clinically relevant; retrospective recall is imperfect). 

 a. The background mentions challenges of prospective menstrual data but does not clearly articulate why existing algorithms or definitions (e.g., STRAW+10) could not be applied directly, or why ALSPAC’s structure necessitates this custom approach.

 b. Limited engagement with prior methodological work on determining menopausal status in longitudinal cohorts

 c. The background references health associations of menopause timing but provides very little discussion of:

 i. how other studies derive menopause age from repeated measures,

 ii. what methodological gaps exist, and

 ii. Lack of explicit problem framing

 2. Methods: The study doesn't account for women with irregular periods

 Overall comments

 This data note describes the development of a reproducible algorithm to estimate age at natural menopause among mothers enrolled in the ALSPAC cohort. The authors synthesise repeated menstrual cycle data collected between 2008 and 2020, combining self-reported last menstrual period (LMP), bleeding patterns in the last 3 and 12 months, and contextual variables such as surgery, contraceptive use, and HRT.

 1. These tolerance thresholds appear somewhat arbitrary. Sensitivity analyses or justification based on empirical patterns in the data would strengthen confidence.

 2. The decision to use the most recent plausible LMP is reasonable but should be more explicitly discussed.

 3.Add a concise description of the final derived variables (names, meanings, coding of method/source, censoring flags).

 4. Briefly summarise missingness patterns in the derived menopause variable and key reasons (e.g. still menstruating at last contact, hormone use, long gaps).

Are sufficient details of methods and materials provided to allow replication by others?

Partly

Is the rationale for creating the dataset(s) clearly described?

Partly

Are the datasets clearly presented in a useable and accessible format?

Yes

Are the protocols appropriate and is the work technically sound?

Yes

Reviewer Expertise:

Women´s health, menopuase, endometriosis

I confirm that I have read this submission and believe that I have an appropriate level of expertise to confirm that it is of an acceptable scientific standard, however I have significant reservations, as outlined above.

# Response

**Published:** 2026-06-08

**Authors:** Rochelle Knight

**Affiliations:** University of Bristol Bristol Population Health Science Institute, Bristol, England, UK

We thank the reviewer for their careful review of this manuscript and for their constructive and helpful comments. In response to all points raised:

1. Rational and methodological background

We have added further detail on the background and rationale of our study. Whilst previous studies have derived the timing of the FMP within ALSPAC, detailed description of the derivation methods were not provided and some only considered data assessed at the clinic questionnaires. The STRAW+10 criteria provide a framework for classifying menopausal stage (e.g., reproductive, perimenopausal, and postmenopausal stages). However, deriving a specific age at menopause from longitudinal menstrual data involves additional methodological considerations, including missing data and conflicting responses, which are not the focus of the STRAW+10 staging system. Similarly, in other cohorts, there is often limited methodological detail on how age at menopause was derived beyond stating that a 12-month period of amenorrhea was identified. The method presented here was developed to provide a transparent and reproducible approach to estimating age at menopause using all the prospectively collected menstrual data available within ALSPAC.

1. Women with irregular cycles

In response to your comments regarding women with irregular cycles, menopause was defined retrospectively based on 12 months of amenorrhea, consistent with standard definitions of natural menopause. Therefore, women with irregular cycles could still be assigned an estimated age at menopause if they subsequently met the criterion of 12 consecutive months without menstruation and did not report another explanation for cessation of periods (e.g., hormonal contraceptive use, HRT use, surgery, chemotherapy, or other medical causes).  

1. Tolerance thresholds

Given the nature of self-reported longitudinal data, some questionnaire responses were conflicting or internally inconsistent. To ensure these cases were handled in the same way, tolerance thresholds were required. These decisions were informed through detailed discussion within the research team and a clinician, consideration of clinical knowledge regarding menopausal timing and menstrual patterns, and examination of patterns within the data. Whilst sensitivity analyses may be informative, implementing multiple alternative derivation thresholds was beyond the scope of the current data note.

1. Use of the most recent plausible LMP

We appreciate this point and have added further discussion of this within the limitations section.

1. Missingness in age at menopause variable

We summarise under ‘Description of the population’ those women who we were unable to derive age at menopause. In the limitations section we discuss the long gaps between assessment timepoints. We thank the reviewer again for these constructive comments, which have helped strengthen the clarity of the manuscript.

# Reviewer report: Reviewer response for version 1

**DOI:** 10.21956/wellcomeopenres.27277.r139244

**Published:** 2025-12-24

**Authors:** Zailing Xing

**Affiliations:** 1 University of South Florida, Tampa, Florida, USA

Thank you for the opportunity to review this manuscript. My comments are as follows:

1. Expectation of Predictive Performance Metrics
  Based on the title, I expected the study to report predictive performance metrics such as the area under the ROC curve (AUC), sensitivity, specificity, and related measures to evaluate the proposed tool/algorithm. These metrics are essential for assessing predictive accuracy, yet they were not presented in the manuscript.
2. Concerns About Predictive Accuracy Using Questionnaire-Based Variables Alone
  I have concerns about whether the variables included in the questionnaire are sufficient to accurately predict future age at menopause. It is unclear that an algorithm based solely on self-reported or survey-based variables would perform reliably in real-world settings. Incorporating biological or laboratory markers—such as sex hormone levels—would likely strengthen the model and improve predictive validity.
3. Tables 2 and 3: Suggested Placement in Supplementary Materials
  Tables 2 and 3 occupy substantial space in the main manuscript but are not central to the interpretation of the primary findings. I recommend moving these tables to the supplementary section to enhance the readability and flow of the main text.

Are sufficient details of methods and materials provided to allow replication by others?

No

Is the rationale for creating the dataset(s) clearly described?

No

Are the datasets clearly presented in a useable and accessible format?

No

Are the protocols appropriate and is the work technically sound?

No

Reviewer Expertise:

Menopause, Women's health

I confirm that I have read this submission and believe that I have an appropriate level of expertise to state that I do not consider it to be of an acceptable scientific standard, for reasons outlined above.

# Response

**Published:** 2026-06-08

**Authors:** Rochelle Knight

**Affiliations:** University of Bristol Bristol Population Health Science Institute, Bristol, England, UK

We appreciate the reviewer’s careful consideration of our work and their helpful feedback. Regarding the point on predictive performance metrics, we would like to clarify that this study is not intended as a predictive model of future age at menopause. Rather, it is a data note describing the derivation of age at menopause using prospectively collected longitudinal menstrual data within ALSPAC. For this reason, predictive performance metrics would not be appropriate to include. Unlike most studies, in which age at menopause is reported retrospectively at a later age, ALSPAC assessed prospective information on women’s menstrual history, i.e., what was the date of your last menstrual period? Using these questions we have estimated the women’s age at menopause based on identifying a period of amenorrhea of more than 12 months and have accounted for factors affecting menstruation such as surgery, contraception, and HRT. We have also dealt with missing data and inconsistent responses. Given your comments, we have revised the manuscript wording to clarify that the approach is intended for estimation of age at menopause from longitudinal data, rather than prediction, and we have adjusted terminology accordingly where needed. We have decided to keep Tables 2 and 3 in the main document.

# Reviewer report: Reviewer response for version 1

**DOI:** 10.21956/wellcomeopenres.27277.r139241

**Published:** 2025-12-23

**Authors:** Madeline Wood Alexander

**Affiliations:** 1 University of Toronto, Toronto, Ontario, Canada

This is an excellent article detailing the algorithm used to ascertain age at menopause in a longitudinal cohort of midlife women with repeated self-report measures of menstrual history and other relevant variables. The authors describe and apply a rigorous method to estimate age at natural menopause, accounting for surgical procedures, use of hormone therapy/hormonal contraceptives, and performing numerous internal consistency checks to produce the most accurate estimates of last menstrual period (LMP) possible.

 The authors note that they censored data at timepoints where participants reported taking hormonal contraceptives or hormone therapy, given that age at menopause cannot be ascertained while an individual is taking these medications. While this decision is justified, the exclusion of these participants from any future analyses of age at menopause may introduce bias. Perhaps this should be noted as a limitation or warning to future researchers. 

 Additionally, it would be beneficial to know how many participants have an available age at surgical menopause, and how the distribution of this variable differs from natural menopause.

 Overall this is a very high-quality contribution that will be an important resource to the scientific community, especially for scientists using the ALSPAC data for women's health research.

Are sufficient details of methods and materials provided to allow replication by others?

Yes

Is the rationale for creating the dataset(s) clearly described?

Yes

Are the datasets clearly presented in a useable and accessible format?

Yes

Are the protocols appropriate and is the work technically sound?

Yes

Reviewer Expertise:

Women's brain health

I confirm that I have read this submission and believe that I have an appropriate level of expertise to confirm that it is of an acceptable scientific standard.

# Response

**Published:** 2026-06-08

**Authors:** Rochelle Knight

**Affiliations:** University of Bristol Bristol Population Health Science Institute, Bristol, England, UK

We are grateful to the reviewer for reviewing this manuscript and for providing helpful comments and suggestions. We have added to the limitations section that the exclusion of timepoints where hormonal medications were reported may introduce bias and that this should be considered by future researchers. Regarding surgical menopause, unfortunately we are unable to reliably assign an age at surgical menopause. Although participants were asked about surgeries related to the removal of reproductive organs, not all reported procedures would necessarily result in surgical menopause. For example, hysterectomy without bilateral oophorectomy will lead to cessation of menstruation but not menopause itself. In addition, some participants reported having had ‘surgery’ that stopped periods without specifying the type of surgery or the timing of the procedure. As a result, deriving a reliable variable for age at surgical menopause would require substantial additional considerations beyond the scope of the current data note. We thank the reviewer again for recognising the potential value of our work to researchers using the ALSPAC data for women’s health research.

# Reviewer report: Reviewer response for version 1

**DOI:** 10.21956/wellcomeopenres.27277.r139243

**Published:** 2025-11-25

**Authors:** Annette Dobson

**Affiliations:** 1 The University of Queensland Integrative Legume Research Group, Saint Lucia, Queensland, Australia

This is an excellent contribution to the literature on age at menopause. It provides detailed documentation of the algorithm used in ALSPAC. This is one of the few studies where age at menopause can be obtained 'prospectively' (for most women) as many studies that have reported age at menopause have recruit women later in life and final menstrual period (FMP) is reported retrospectively and subject to recall bias.

 The authors provide comprehensive detail of how they calculate the date of FMP from questions that were not worded or administered consistently over time - a common situation in longitudinal studies where the main focus was not on menstruation.

 They mention there are particular difficulties with women who report taking the oral contraceptive pill, HRT, or have had surgery or other conditions or treatments that disrupt natural menstruation. They 'censor' these women but do not describe how they calculate dates of censoring which will be necessary for any survival-type analyses. Such additions would enhance the paper.

Are sufficient details of methods and materials provided to allow replication by others?

Yes

Is the rationale for creating the dataset(s) clearly described?

Yes

Are the datasets clearly presented in a useable and accessible format?

Yes

Are the protocols appropriate and is the work technically sound?

Yes

Reviewer Expertise:

Biostatistics, women's health

I confirm that I have read this submission and believe that I have an appropriate level of expertise to confirm that it is of an acceptable scientific standard.

# Response

**Published:** 2026-06-08

**Authors:** Rochelle Knight

**Affiliations:** University of Bristol Bristol Population Health Science Institute, Bristol, England, UK

We thank the reviewer for taking the time to review this manuscript and for their thoughtful comments. In response to your comment regarding censoring dates, we realise that our use of the term “censor” may have been unclear. In the context of our work, women were not censored in a formal survival-analysis sense. Rather, reported dates of last menstrual period (LMP) from timepoints where factors likely to affect natural menstrual bleeding were reported (e.g., hormonal contraceptive use, HRT use, surgery, chemotherapy, or other conditions affecting menstruation) were excluded from consideration when estimating the final menstrual period (FMP). In practice, this was determined using information reported at each questionnaire timepoint. For example, if a woman reported both a date of LMP and current hormonal contraceptive use at a given questionnaire, the reported menstrual date from that timepoint was excluded because hormonal contraceptives may alter bleeding patterns. If hormonal contraceptive use was no longer reported at a later questionnaire, menstrual information from that subsequent timepoint could again contribute to estimation of the FMP. Following this comment, we have clarified this process in the manuscript and replaced the term “censor” with “exclude” throughout to avoid confusion with censoring in survival analyses.
